# Src Activation Aggravates Podocyte Injury in Diabetic Nephropathy *via* Suppression of FUNDC1-Mediated Mitophagy

**DOI:** 10.3389/fphar.2022.897046

**Published:** 2022-05-09

**Authors:** Ting Zheng, Han-yu Wang, Yang Chen, Xiao Chen, Zi-ling Wu, Qin-yu Hu, Hui Sun

**Affiliations:** Department of Endocrinology, Union Hospital, Tongji Medical College, Huazhong University of Science and Technology, Wuhan, China

**Keywords:** diabetic nephropathy, mitophagy, FUNDC1, Src, phosphorylation

## Abstract

**Background and purpose:** Mitophagy plays a significant role in the progression of diabetic nephropathy (DN), although the regulatory mechanisms remain unclear. Recently, accumulating evidence demonstrated that impaired mitochondrial function and mitophagy are involved in DN. Here, we are aimed to explore the role of c-Src (Src) and FUNDC1-related mitophagy in the development of DN.

**Methods:** The db/db mice were used to establish a DN mice model. The mice accepted PP2 (Src inhibitor) treatment to study the role of Src in DN. Kidney function was measured *via* biochemical testing. Renal histopathology and morphometric analysis were conducted *via* hematoxylin-eosin (HE), periodic acid-Schiff (PAS), Masson’s staining, and transmission electron microscopy (TEM). We measured degree of apoptosis in kidney by TUNEL assay. Indices of mitophagy (LC3 and p62) were evaluated by Western blotting and immunofluorescence. Complementary *in vitro* assays were conducted using human podocytes subjected to high glucose in combination with PP2 treatment or FUNDC1 small interfering RNAs (siRNAs). Flow cytometry was used to detect the apoptotic cells. Mitochondrial function was evaluated by JC-1 staining. Double immunofluorescence labeling of LC3 and TOMM20 used to assess the degree of mitophagy.

**Results:** Increased Src activation was detected in the kidneys of db/db mice, and its expression was positively correlated with mitochondrial damage, podocyte apoptosis, and renal dysfunction. Inhibition of Src activation with PP2 protected against mitochondrial damage and podocyte apoptosis. *In vitro* experiments in podocytes established that high glucose increased Src activation, promoting FUNDC1 phosphorylation and inhibiting mitophagy. Consistent with the mouse model, inhibiting Src activity protected podocytes against mitochondrial damage. FUNDC1 silencing negated the actions of PP2, indicating that FUNDC1-mediated mitophagy is downstream pathway of Src.

**Conclusion:** In summary, our data indicated that Src is a culprit factor in diabetic renal damage *via* suppression of FUNDC1-mediated mitophagy, promoting the development of DN.

## Introduction

Diabetic nephropathy (DN) is one of the most common causes of end-stage renal disease (ERSD) (Umanath and Lewis, 2018). Approximately 40% of diabetes mellitus type 2 (T2DM) patients will develop DN, making this condition a major microvascular complication of diabetes mellitus (DM) (Lu et al., 2017). With the increasing morbidity and mortality resulting from DN worldwide, a serious and urgent public health issue is emerging (Maqbool et al., 2018). Thus, a better understanding of pathogenesis of DN is expected to pave the way for novel clinical treatments and improve outcomes for affected patients.

Podocytes represent an indispensable component of the glomerular filtration barrier with previous studies highlighting that podocytes undergo structural and functional changes during the earliest stages of DN pathogenesis (Susztak et al., 2006). Indeed, podocyte injury plays a central role in DN development, and their levels of damage correlate with the degree of renal impairment (Torban et al., 2019). Although the cellular and molecular mechanisms underlying DN development remains unclear, substantive evidence points to the contribution of mitophagy to the development of DN (Saxena et al., 2019; Sun et al., 2019; Zhou et al., 2019).

Mitophagy is the specific form of autophagy that selectively removes damaged or defective mitochondria (Wang Y. et al., 2020). Currently, two different mitophagy pathways have been discovered, namely the PINK/Parkin pathway and the receptor-mediated pathway (Palikaras et al., 2018). Regarding the latter, a range of different mitophagy receptors including NIX (Novak et al., 2010; Yuan et al., 2017), BCL2L13 (Murakawa et al., 2015; Otsu et al., 2015), FUNDC1 (Wang J. et al., 2020), BNIP3 (Hanna et al., 2012; Jin et al., 2018; Shi et al., 2018), and PHB2 (Lahiri and Klionsky, 2017; Wei et al., 2017; Yan et al., 2020; Zhang et al., 2020) contain an LC3-interacting region (LIR) that recruits MAP1LC3B/Atg8-phagophore membranes for identifying and devouring defective mitochondria. Notably a recent study highlighted the role of Parkin-mediated mitophagy in the pathogenesis of DN (Sheng et al., 2018). Here, NR4A1 knockdown increased Parkin-mediated mitophagy, reducing hyperglycaemia mediated mitochondrial damage and thus improving renal function (Sheng et al., 2018). Nevertheless, different mitophagy pathways have diverse impacts on cell fates and it is likely that a range of mitophagy pathways are involved in DN pathogenesis.

Among these pathways, FUNDC1-mediated mitophagy provides known advantages for cell survival. For example, FUNDC1-related mitophagy benefits the survival of the microvasculature following ischemia reperfusion (IR) injury (Zhang et al., 2017; Zhou et al., 2018). Insufficient FUNDC1-mediated mitophagy induces mitochondrial debris formation, evokes mitochondrial oxidative stress, impairs mitochondrial respiratory function, leading to mitochondrial-induced apoptosis (Zhou et al., 2017a; Zhou et al., 2017b). Activation or inactivation of FUNDC1 is regulated *via* phosphorylation (Wang and Zhuang, 2017). Phosphorylated form of FUNDC1 act to restrain the induction of mitophagy by preventing the FUNDC1 LIR from interacting with LC3 (Chen et al., 2017). Therefore, phosphorylation is critical for regulating FUNDC1-related mitophagy.

Src is the prototypical member of the Src family kinases, a small family of nonreceptor protein tyrosine kinases implicated in the regulation of numerous cellular processes, including cell proliferation, differentiation, migration and invasion, and angiogenesis (Taniguchi et al., 2013). Recently, overwhelming evidence has emerged linking Src to the development of DN (Chen et al., 2015) although the underlying mechanisms remain incompletely understood. Of relevance to this study, it has been previously reported that the Src kinase acts as a negative regulator of mitophagy by phosphorylating FUNDC1 at Tyr 18 (Liu et al., 2012). Presently, it has not been ascertained whether FUNDC1-mediated mitophagy contributes to DN. Based on these findings, we hypothesized that the effect of Src on DN involves its regulation of FUNDC1-mediated mitophagy. Thus, we explored the role of Src and FUNDC1 in the progression of DN.

## Materials and Methods

### Animals

Male db/db mice (*n* = 10) and db/m mice (*n* = 5) were purchased from Gem Pharmatech Co., Ltd. At 8 weeks of age, the mice were allocated to either the db/m group (control, *n* = 5), db/db group (*n* = 5) or PP2 group (PP2-treated db/db mice, *n* = 5). PP2 is an inhibitor of Src, which inhibits Src activation. In the PP2 group, mice were treated *via* intraperitoneal injection with PP2 (2 mg/kg, Topscience, China) every other day while an equal volume of normal saline and DMSO was administered intraperitoneally to mice in the db/m and db/db groups. All animals were euthanized at 16 weeks. Blood and kidney tissue collected for biochemical tests and pathological assessment, respectively. The animal protocols used in this study were approved by the Animal Ethics Committee of Huazhong University of Science and Technology (IACUC Number: 2462).

### Renal Tissue Morphological Analysis

After rapid isolation, kidney tissues were fixed with 4% formaldehyde and embedded in paraffin or fixed in 2.5% glutaraldehyde as required. Histological staining was conducted with HE, PAS, and Masson’s trichrome to observe pathological changes. Kidney ultrastructure was examined by TEM. NIH ImageJ software (National Institutes of Health) was used to analyse TEM images of kidney tissues, including the thickness of the glomerular basement membrane (GBM), foot process width, and the number of foot processes/µm. Images of TEM were analyzed as previously described (Chen et al., 2020).

### Cell Culture

Human conditionally immortalized podocytes were cultured at 33°C in RPMI medium (HyClone, United States) supplemented with 10% fetal bovine serum (Gibco, Australia), insulin-transferrin-selenium (ITS) (Gibco, United States), 100 μg/ml streptomycin and 100 U/mL penicillin G (Gibco, United States) in a 5% CO_2_ atmosphere. For differentiation, the cells were transferred to ITS-free medium at 37°C for 7–10 days. Thereafter, the differentiated cells were treated with a normal glucose concentration (NG) (5 mM), hypertonic solution (MA, NG combined with 25 mM mannitol), or high glucose concentration (HG) (30 mM) for 48 h, respectively. As indicated, Src inhibitor PP2 (2 uM) was added 2 h before exposure to HG.

### Transfection

Podocytes were transfected with siRNAs targeting FUNDC1 or NC control (GenePharma) according to the manufacturer’s protocol. Briefly, podocytes were seeded into six-well plates at a density of 2 × 10^5^ cells/well and transfected for 2 days with FUNDC1 siRNA using the Lipofectamine 2000 reagent (Invitrogen). The cells were next incubated under normal conditions at 37°C.

### Apoptosis Assay

The degree of podocyte apoptosis *in vitro* was assessed by flow cytometry with Annexin V-APC/7-AAD double staining according to the manufacturer’s instructions (Annexin V-APC/7-AAD Double Staining Cell Apoptosis Detection Kit, KEYGENE, China).

### TUNEL Assay

The TUNEL kit (*In Situ* Cell Death Detection Kit, Fluorescein, Roche) was used to detect apoptosis in kidney tissues. Briefly, tissue sections were deparaffinized and hy-drated before treating with Proteinase K (Roche) for 15–30 min at 37°C. After washing three times with PBS for 5 min each, TUNEL reaction solution (50 ul) was added to the sections for 60 min. The TUNEL reaction solution was prepared according to the ratio of solution A (Enzyme Solution 10x): Solution B (Label Solution) = 1:9. DAPI was used to counterstain nuclei and TUNEL-positive cells were counted using Image J.

### Western Immunoblotting

Cells lysates were prepared using RIPA buffer (Beyotime, China) containing PMSF and phosphatase inhibitor (Servicebio, China). The extracts were clarified by centrifugation at 12,000 rpm for 10 min at 4°C and total protein concentrations estimated using the BCA Protein Assay Kit (Beyotime, China). Equal protein amounts were prepared in SDS-PAGE loading buffer and heated at 100°C for 5 min before protein separation by SDS PAGE and transfer to PVDF membranes (Millipore). After blocking with skim milk solution for 90 min, membranes were incubated with the indicated primary antibodies**.** (Src rabbit monoclonal antibody, 1:1000, CST; Phospho-Src Family (Tyr416) (D49G4) rabbit monoclonal antibody, 1; 1000, CST; t-FUNDC1 rabbit polyclonal antibody, 1:500, abcepta; p-FUNDC1 (Tyr18) rabbit polyclonal antibody, 1:500, abcepta; LC3 rabbit polyclonal antibody, 1:1000, Proteintech; P62 rabbit polyclonal antibody, 1:1000, CST; GAPDH Mouse monoclonal antibody, 1:3000, Antgene, China; Detailed antibody information is provided in Supplementary Table S1) followed by the appropriate anti-rabbit or mouse HRP-conjugated secondary antibodies. The membranes were incubated with ECL solution and protein bands visualized in an imaging system. The intensities of bands were quantified using Image J software. Uncropped Western blot images are provided in Supplementary Figure S1.

### Mitochondrial Membrane Potential Assay

Decreases in MMP represent a hallmark event of early apoptosis (Marchi et al., 2018) and cellular changes in MMP were analyzed using the MMP assay kit (Beyotime, China) employing JC-1 staining. In the normal state, JC-1 aggregates in the mitochondrial matrix to form polymers, producing red fluorescence signals; when mitochondrial membrane potentials decrease, JC-1 disaggregates to monomers which produce green fluorescence. Assays were conducted as follows. After washing with PBS, the cell monolayers were stained with JC-1 for 30 min at 37°C before washing with binding buffer. Next, cell photomicrographs were recorded using epifluorescence microscopy. The data presented as the ratio of red and green fluorescence after averaging the fluorescence intensity values of each cell using Image-Pro Plus 6.0 software.

### Immunofluorescence

Kidney tissue sections were deparaffinized and hydrated before being subjected to antigen retrieval. The sections were then incubated with 10% donkey serum for 30 min at room temperature before addition of primary antibodies against nephrin (guinea pig polyclonal antibody, Progen, 1:100) and LC3 (rabbit polyclonal antibody, Abcam, 1:200) at 4°C overnight. The next day, sections were washed 3 times, 5 min each time with TBST before adding the appropriate fluorophore-conjugated secondary antibody for 1 h in the dark. Nuclei were counterstained with DAPI (Beyotime, China) for 10 min and the sections mounted in anti-fade reagent before observation with a confocal microscope. Alternatively, podocytes were fixed on slides with 4% formaldehyde for 15 min, permeabilized with 0.5% Triton X-100 for 20 min and blocked with normal donkey serum for 1 h at room temperature. Thereafter, the cell slides were incubated with LC3 rabbit polyclonal antibody (1:200, Abcam) and TOMM20 mouse monoclonal antibody (1:100; Santa Cruz Biotechnology) overnight at 4°C, washed and further incubated with Alexa Fluor 594 Donkey anti-rabbit IgG (H + L) (1:100, Antgene, China) and Alexa Fluor 488 Donkey anti-mouse IgG (H + L) (1:100, Antgene, China) at room temperature in the dark for 60 min. Nuclei were counterstained with DAPI (Beyotime, China) for 10 min and imaged using an epifluorescence microscope.

### Statistical Analysis

For quantitative analysis, values were acquired from 3 independent experiments and shown as the mean ± SD. Statistical analyses were performed using GraphPad Prism 8 software with statistically significant differences determined by the students t test or one-way ANOVA analysis of variance (*p* values <0.05 indicated significant differences).

## Results

### Effects of PP2 on Renal Function and Pathological Characteristics in Kidneys of Db/Db Mice

**FIGURE 1:**
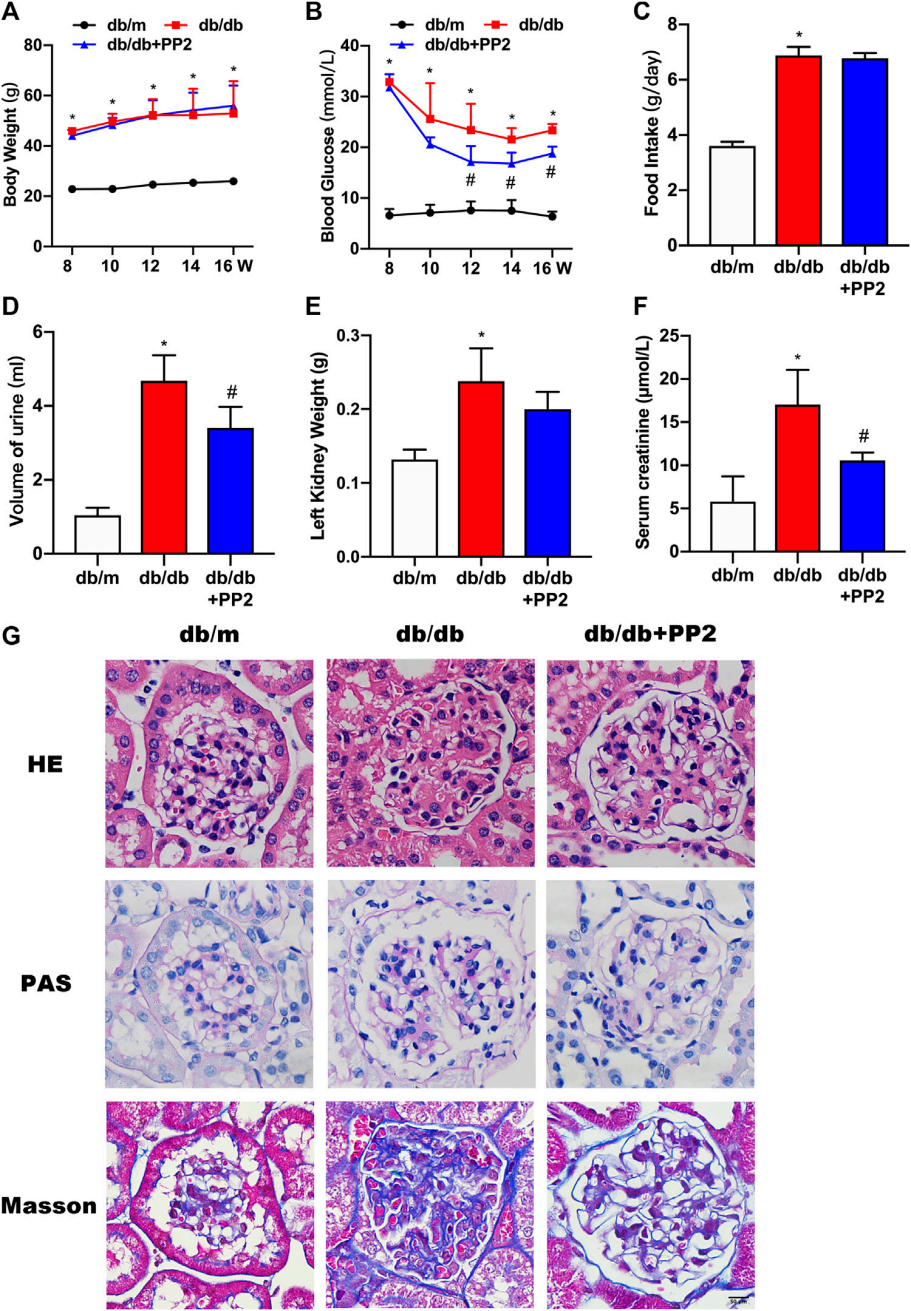
Effects of the Src inhibitor PP2 on the biochemical and histopathological characteristics of db/db mice. **(A)** Body weight changes in the control (db/m) mice, diabetic (db/db) mice and PP2-treated db/db mice from 8 to 16 weeks of age. **(B)** Blood glucose levels in each group. **(C)** Food intake. **(D)** Urine volumes in 24 h. **(E)** Left kidney weights. **(F)** Serum creatinine levels. **(G)** HE, PAS and Masson’s staining were used to evaluate the histological changes and glomerulus damage in each treatment group (Magnification, 400 ×). Values are expressed as the mean ± SD, ^*^ *p* < 0.05 vs. db/m group; ^#^ *p* < 0.05 vs. db/db group. *n* = 5.

As expected, db/db mice displayed higher food intake, increased body and kidney weights, increased blood glucose and serum creatinine levels and produced more urine than db/m mice (Figures 1A–F). Db/db mice treated with the Src inhibitor PP2 displayed decreased levels of blood glucose and serum creatinine compared to db/db mice receiving control treatment (Figures 1B,F). However, PP2 treatment produced no significant changes in body and kidney weights in db/db mice (Figures 1A,E). Further assessment of kidney morphology by HE and PAS staining showed that glomerular hypertrophy and proliferation of the mesangial matrix in db/db mice and Masson’s staining showed evidence of glomerular fibrosis (Figure 1G). Notably, PP2 treatment produced dramatic reductions in these pathological changes in the db/db mice (Figure 1G) suggesting a protective effect on kidney function in diabetic mice.

### Inhibition of Src Activity Ameliorates Podocyte Injury

**FIGURE 2:**
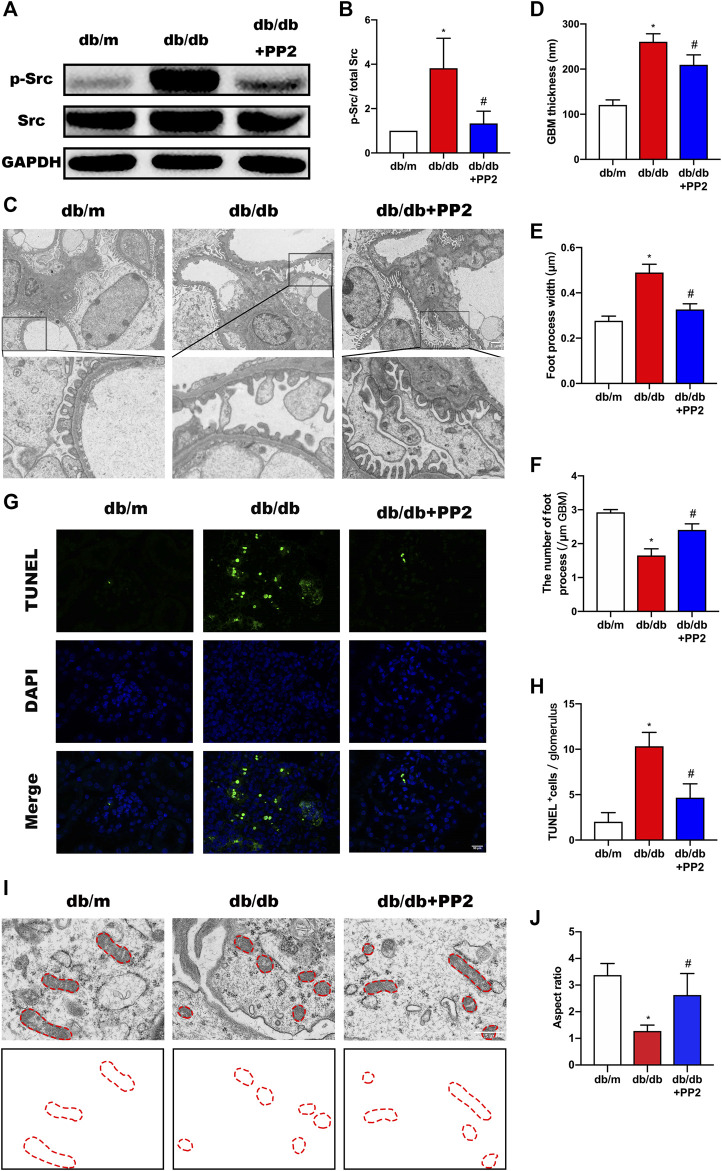
Inhibition of Src activity ameliorates podocyte injury. **(A)** Representative Western blot images of Src and p-Src levels in kidney tissues from the different treatment groups. **(B)** Quantification of expression levels of Src and p-Src; GAPDH was used as the loading control. **(C)** Representative transmission electron micrographs showing changes in podocyte in the kidneys of the different groups. (Magnification, 3000 ×). **(D–F)** Quantitation of the thickness of the glomerular basement membrane (GBM), foot process widths and number of foot processes/µm. **(G)** TUNEL assays evaluating cell apoptosis in glomeruli among the different groups. TUNEL-positive (apoptotic) cells are stained green, and nuclei labeled with DAPI (blue). (Magnification, 400 ×). **(H)** Quantitative calculation of number of TUNEL-positive cells in each glomerulus. **(I)** Representative electron micrographs of mitochondrial ultrastructure in podocytes from each treatment group. (Magnification, 12,000 ×), **(J)** Quantitative calculation mitochondrial aspect ratio in each group. Values are expressed as the mean ± SD, ^*^ *p* < 0.05 vs. db/m group; ^#^ *p* < 0.05 vs. db/db group. *n* = 5.

The activation state of Src can be estimated from its phosphorylation state. Western blotting analysis showed that the levels of phosphorylated (p)-Src were increased in kidneys from db/db mice compared with db/m mice (Figures 2A,B). Notably, along with hyperglycemia, PP2 treatment resulted in reductions in the levels of p-Src in db/db kidney tissues, providing evidence that PP2 inhibited Src activation *in vivo*. Furthermore, transmission electron microscopy (TEM) analysis of kidney tissues showed the exacerbated podocyte injury apparent in db/db mice was reversed by PP2 treatment. There were reductions in glomerular basement membrane (GBM) thickening and broadening of podocyte foot processes after PP2 treatment (Figures 2C–F). Moreover, the number of apoptotic cells in the glomeruli of db/db mice were also reduced by PP2 treatment (Figures 2G,H). To further observe mitochondrial damage in podocytes, we used TEM to analyze the morphology of podocyte mitochondria in each group. The results showed that the mitochondrial fission of podocytes in db/db mice was significantly increased compared with db/m mice. However, db/db mice administered PP2 treatment had reduced fission of podocyte mitochondria (Figures 2I,J). Collectively these data indicate that Src activation underlies the pathological changes occurring in the diabetic mouse kidney and Src inhibition can ameliorate podocyte injury associated with compromised mitochondrial integrity.

### Activation of Src Represses FUNDC1-Mediated Mitophagy in DN

**FIGURE 3:**
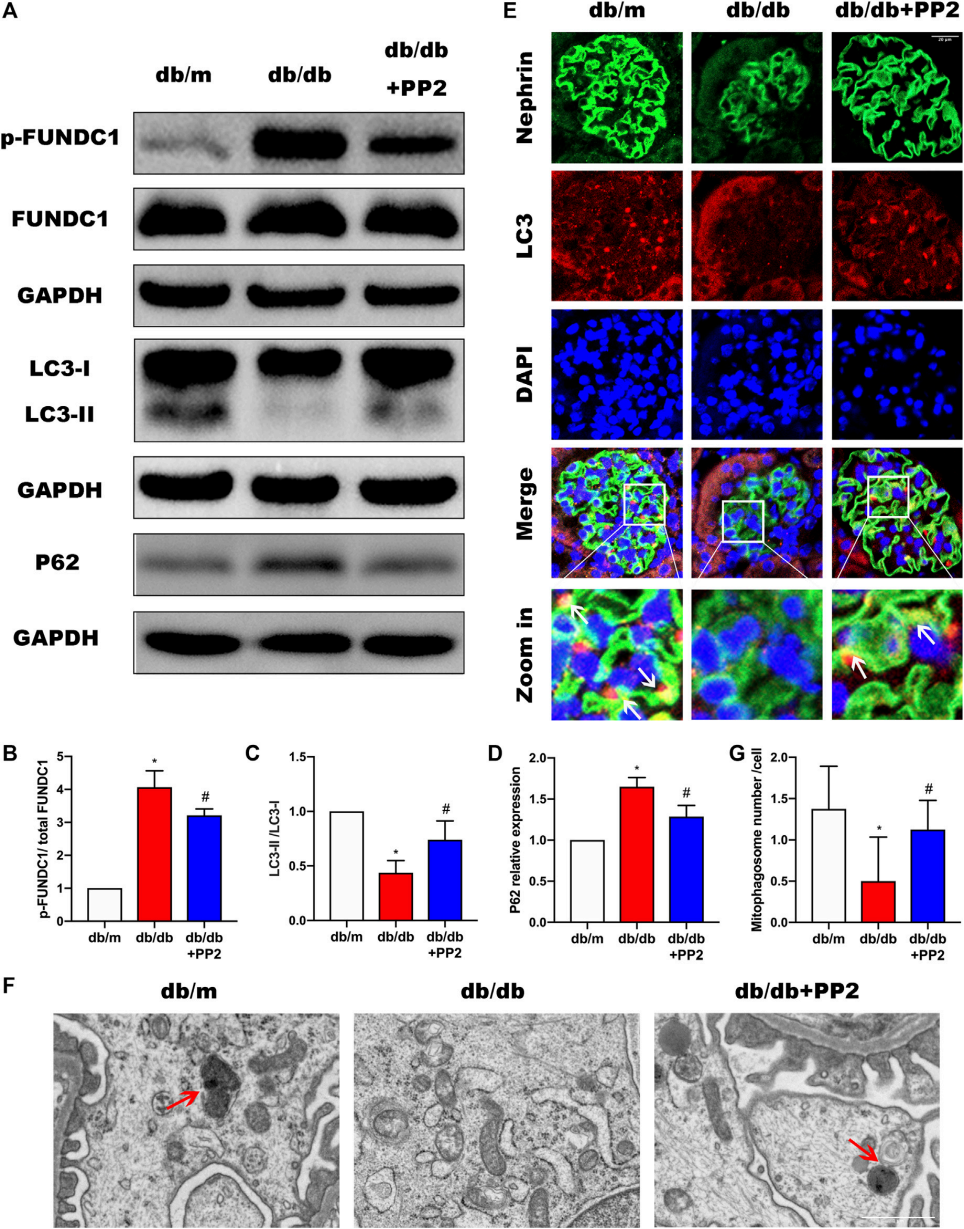
Inhibition of Src activity increases mitophagy during DN. **(A)** The expression levels of FUNDC1, p-FUNDC1, LC3, P62 in kidney tissues from the different groups were analyzed by Western blot. **(B–D)** Quantitative analysis of gray values of bands from Western blots, GAPDH was used as loading control. **(E)** Representative images of LC3 (red) staining in glomeruli in kidney tissues from the different groups. Podocytes were marked by Nephrin staining (green). (Magnification, 600 ×). **(F)** Representative TEM images demonstrating mitophagosomes (mitophagosomes engulfing mitochondria) in podocytes from the different groups. Red arrows denote typical mitophagosomes. (Magnification, 5000 ×). **(G)** Quantification of mitophagosomes numbers from the experiment in **(F)**. Values are expressed as the mean ± SD, ^*^ *p* < 0.05 vs. db/m group; ^#^ *p* < 0.05 vs. db/db group. *n* = 5.

High glucose (HG) conditions have been shown cause deficiencies in mitophagy, resulting in mitochondrial dysfunction and apoptosis induction. However, the specific regulatory mechanisms and pathways have not yet been thoroughly studied. We therefore sought to determine if the involvement of Src in the pathogenesis of DN was related to effects on mitophagy. It has been reported that the conversion of LC3I to LC3II may represent autophagosome formation, and the LCII/LC3I ratio has been used autophagic flux (McLeland et al., 2011; Xu et al., 2016). Therefore, to measure changes, mitophagy indices including LC3 along with P62 were evaluated by Western blotting. Comparing the levels of P62 and LCII/LC3I ratio in the kidneys from the different treatment groups, we observed that P62 expression was up-regulated whereas the LCII/LC3I ratio was down-regulated in the kidneys of db/db mice (Figures 3A,C,D). Moreover, we found that LC3 accumulation in podocytes of db/db mice was significantly reduced (Figure 3E). Structural assessment by TEM demonstrated reduced number of mitophagosome vacuoles (Figures 3F,G) indicative of suppression of mitophagy. Interestingly, treating db/db mice with PP2 reversed these changes, indicating that inhibition of Src by PP2 promotes mitophagy. Further measurements of the mitophagy receptor FUNDC1 showed that PP2 suppressed the expression of phosphorylated (p)-FUNDC1 although total (t)-FUNDC1 protein levels were not affected (Figure 3B). Since FUNDC1-mediated mitophagy is inhibited by its phosphorylation, these data suggest that the Src inhibited mitophagy may occur by increasing the levels of p-FUNDC1.

### Activation of Src can Inhibit HG-Induced Mitophagy in Podocytes

**FIGURE 4:**
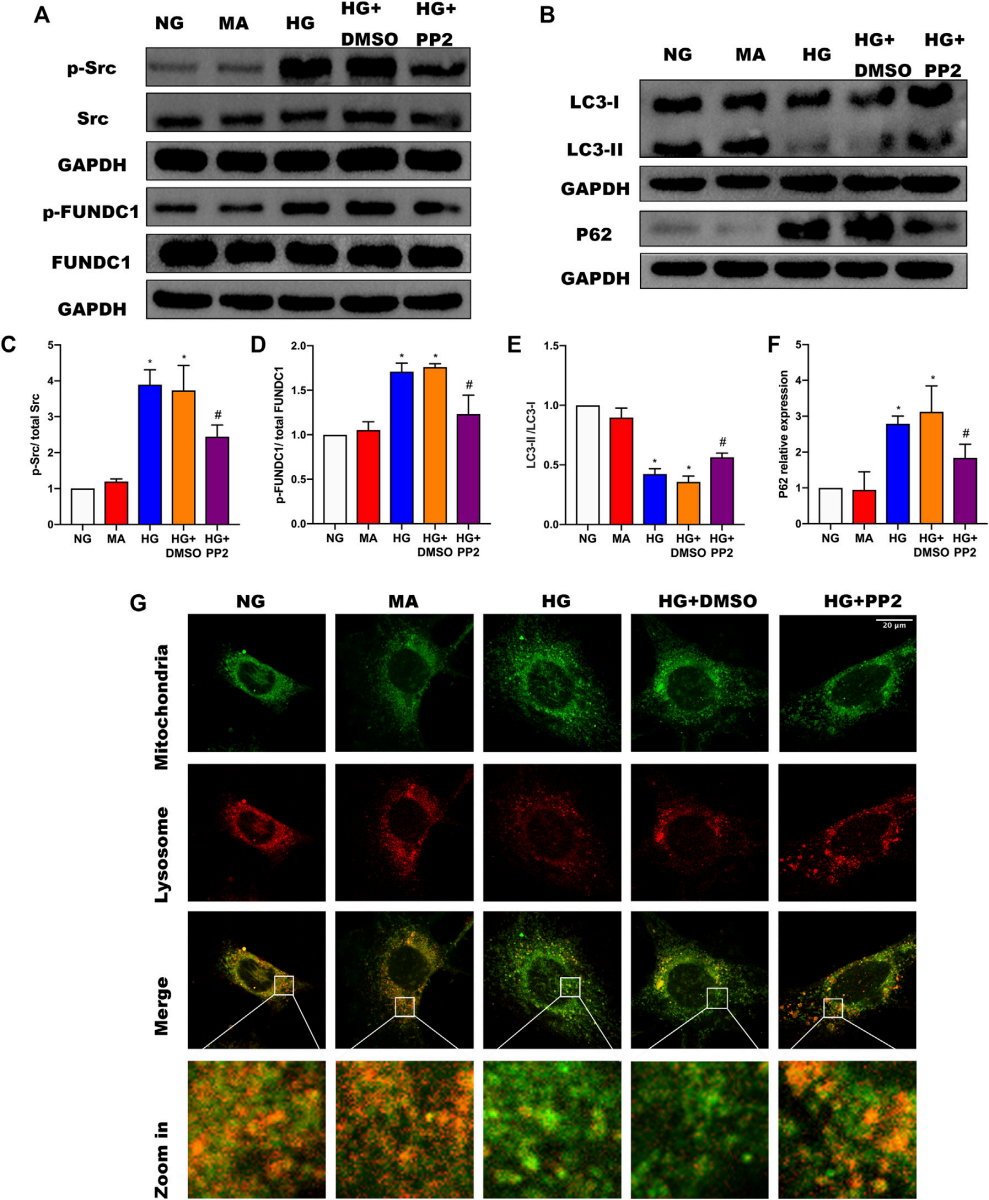
Src activation was upregulated in response to high glucose and inhibits FUNDC1-mediated mitophagy in human podocytes. **(A)** Representative Western blot images of Src, p-Src, FUNDC1 and p-FUNDC1 expression. **(B)** LC3 and P62 expression in podocytes after the indicated treatments. **(C–F)** Quantification of Western blot band intensities of the different proteins from **(A,B)**; GAPDH was used as loading control. **(G)** Co-immunofluorescence staining for mitochondria and lysosome to observe changes in mitophagy in podocytes after the indicated treatments. (Magnification, 1000 ×) Co-localization of mitochondria and lysosome indicated the occurrence of mitophagy. The fragmented mitochondria could not be engulfed by lysosomes after HG treatment, as illustrated by only the sporadic co-localization of mitochondria and lysosome, indicating that mitophagy was inhibited. These changes were reversed by PP2 treatment, which facilitated fusion of mitochondria and lysosomes.NG, normal glucose; MA, mannitol; HG, high glucose. Values are expressed as the mean ± SD, ^*^ *p* < 0.05 vs. NG group, ^#^ *p* < 0.05 vs. HG group.

To address the mechanism whereby activation of Src can regulate mitophagy, we turned to a cultured podocyte model. As anticipated, exposing podocytes to HG conditions resulted in notable increases in p-Src and p-FUNDC1 together with inhibition of mitophagy represented by decreased LCII/LC3I ratio and increased P62 expression (Figures 4A–F). Moreover, inhibiting Src phosphorylation with PP2 reduced phosphorylated FUNDC1 levels and reduced the extent of changes in mitophagy markers. To verify the changes in mitophagy, we co-stained mitochondria and lysosomes to observe mitochondrial-lysosome fusion events. Compared with the NG group, we found that HG caused more defective mitochondria, which could not be captured by lysosomes. Conversely, inhibiting Src activation with PP2 promoted mitophagy (Figure 4G). Together these data suggest that HG disturbs mitochondrial homeostasis through Src-regulated inhibition of FUNDC1-mediated mitophagy.

### Src Activation Is Related to Podocyte Mitochondrial Apoptosis

**FIGURE 5:**
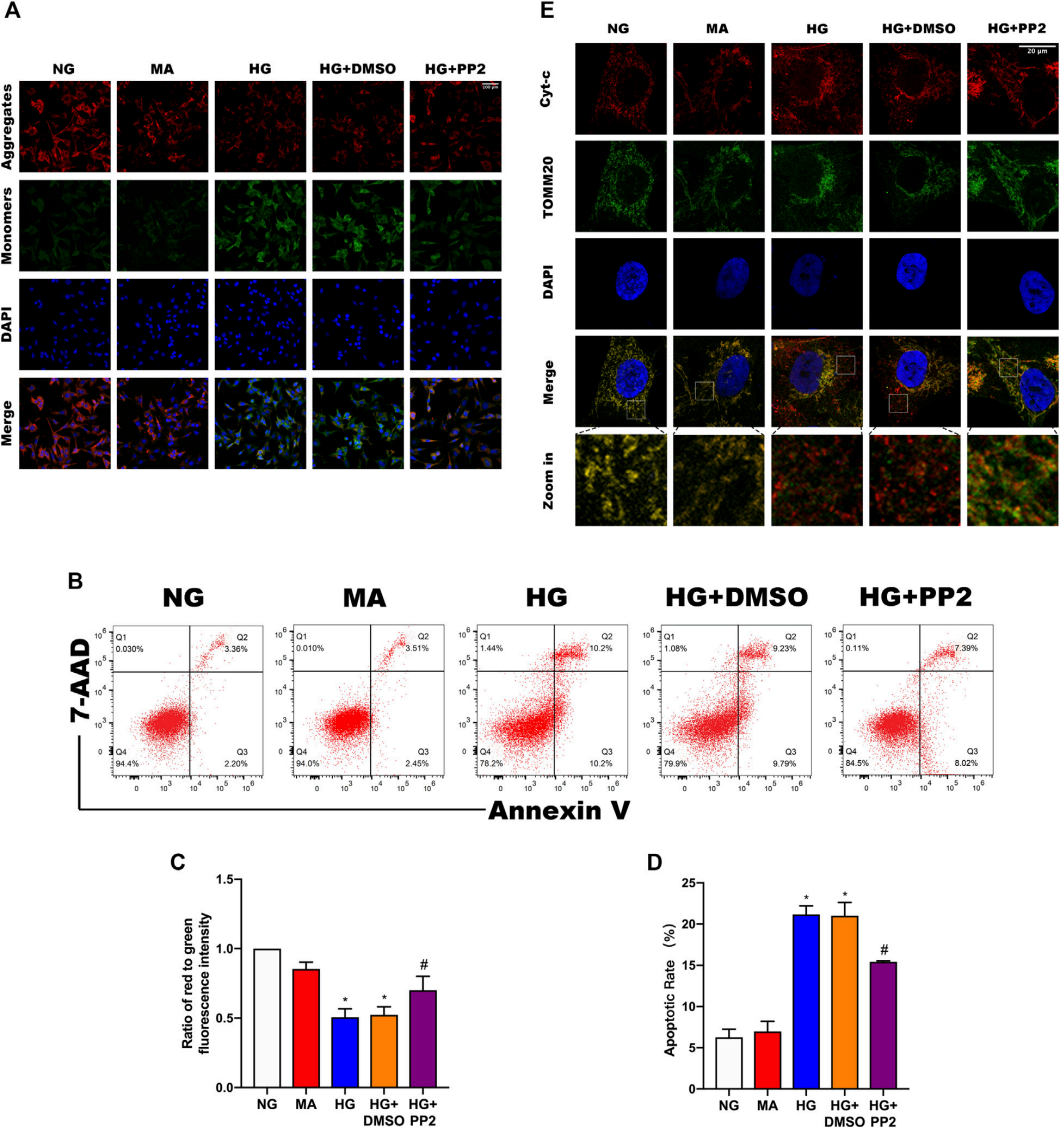
Src activation is related to podocyte mitochondrial apoptosis. **(A)** JC-1 staining to observe mitochondrial membrane potential changes in podocytes after the indicated treatments. Red fluorescence represents normal, functioning mitochondria while green fluorescence indicates collapsed mitochondrial membrane potential. (Magnification, 200 ×). **(B)** Flow cytometric detection of podocyte apoptosis rates. **(C)** MMP measured as the ratio of red to green fluorescence intensity from **(A)**. **(D)** Quantitation of podocyte apoptosis from **(B)**. **(E)** Double immunofluorescence labeling of Cyt-c (red) and TOMM20 (green) in podocytes, nuclei labeled with DAPI (blue) (Magnification, 1000 ×). Values are expressed as the mean ± SD, *p < 0.05 vs. NG group, ^#^ *p* < 0.05 vs. HG group.

Reduction of mitochondrial membrane potential (MMP) is a characteristic manifestation of mitochondrial apoptosis (Marchi et al., 2018). To better understand the link between glucose exposure, Src activation and podocyte damage leading to apoptosis, we assessed MMP levels and apoptosis in podocytes. Notably, exposing podocytes to HG caused decreased both MMP levels (Figures 5A,C) and podocyte apoptosis (Figures 5B,D). However, treating podocytes with PP2 alleviated above changes. Lastly, we confirmed the mitochondrial changes using double immunofluorescence labeling of cells comparing cytochrome c (Cyt-c) which is released from mitochondria during the early stages of apoptosis, and TOMM20, a mitochondrial outer membrane protein that labels mitochondria. Analysis of the comparative distribution of Cyt-c in podocytes showed the characterized punctate appearance of Cyt-c in mitochondria under NG conditions. In contrast, HG promoted Cyt-c leakage from the mitochondria to the cytoplasm and even some nuclear transfer, whereas PP2 treatment largely alleviated the effects of HG treatment (Figure 5E). Together these data indicate that inhibition of Src activation with PP2 reversed the pathological changes in podocytes caused by HG, restoring MMP and consequently reducing podocyte apoptosis.

### Silencing of FUNDC1 in Podocytes Suppressed the Mitophagy Induced by PP2

**FIGURE 6:**
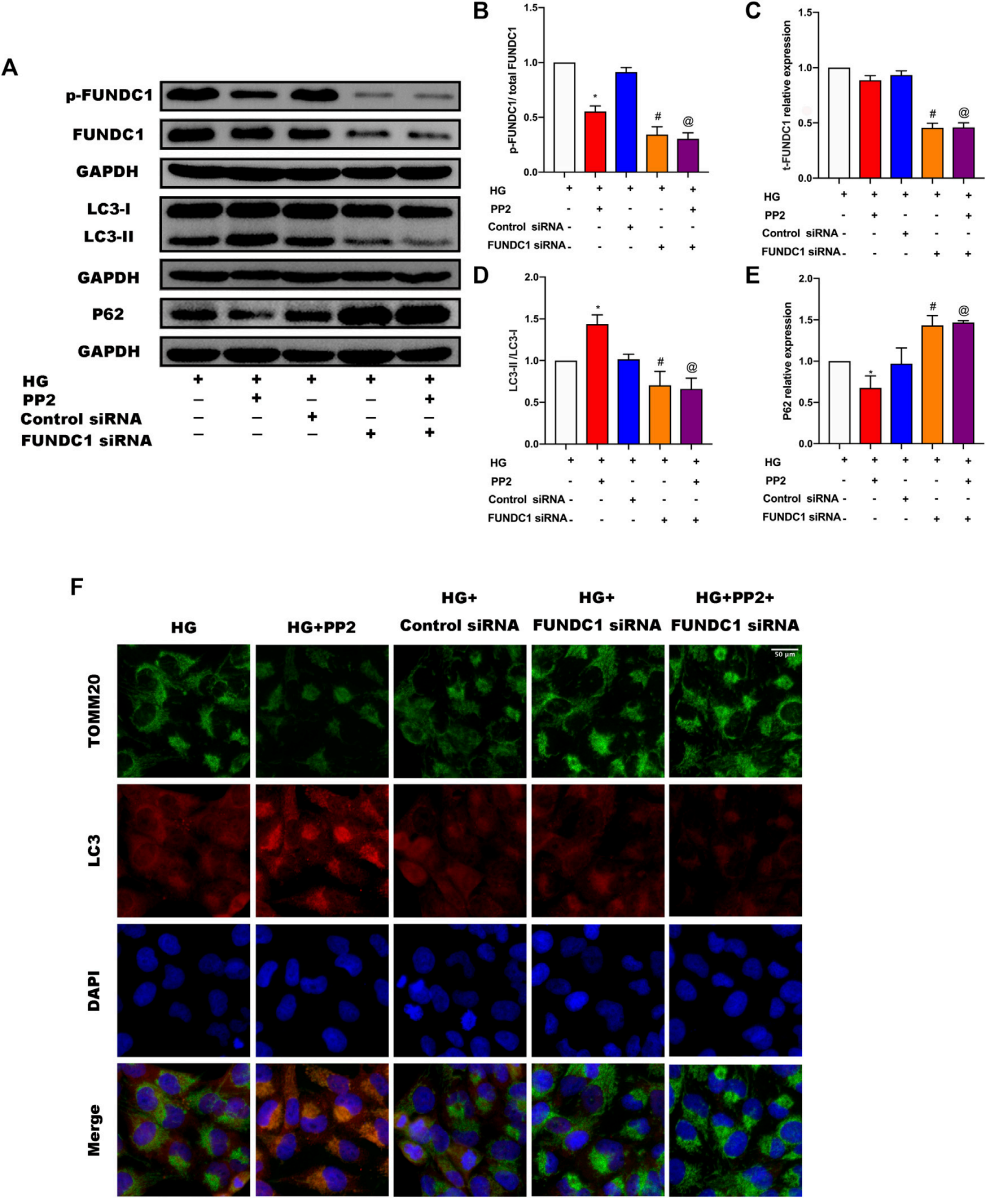
Silencing of FUNDC1 in podocytes suppressed the mitophagy induced by PP2. **(A)** Representative Western blot images of FUNDC1, p-FUNDC, LC3, and P62 expression in podocytes after the indicated treatments. **(B–E)** Quantification of Western blot band intensities of the different proteins; GAPDH was used as loading control. **(F)** Double immunofluorescence staining of LC3 (red) and TOMM20 (green) in podocytes after the indicated treatments (Magnification, 400 ×). Values are expressed as the mean ± SD, *p < 0.05 vs. HG group, ^#^ *p* < 0.05 vs. HG + Control siRNA group, ^@^ *p* < 0.05 vs. HG + PP2 group.

The preceding experiments established a compelling association between Src activation and the suppression of mitophagy. Activation of Src correlated with inactivation of FUNDC1 but as mitophagy initiation can involve some different mitophagy receptors, it was important to establish the contribution of FUNDC1 to podocyte damage. To achieve this, we inhibited the expression of FUNDC1 in podocytes by transfection of FUNDC1 siRNA along with control siRNA (Figures 6A–C). After silencing FUNDC1 we observed reduction in the expression levels of LCII/LC3I ratio and increases in the expression of P62 (Figures 6A,D,E). Double immunofluorescence labeling of LC3 and TOMM20 in podocytes confirmed these findings (Figure 6F). Interestingly, PP2 failed to increase mitophagy in podocytes after transfection with FUNDC1 siRNA. Moreover, compared to control siRNA transfected podocytes, FUNDC1-depleted podocytes were more susceptible to HG-mediated reductions in mitophagy. These results showed that FUNDC1-mediated mitophagy is a major down-stream target deactivated by Src.

### Silencing of FUNDC1 in Podocytes Prevents the Protective Effects of PP2

**FIGURE 7:**
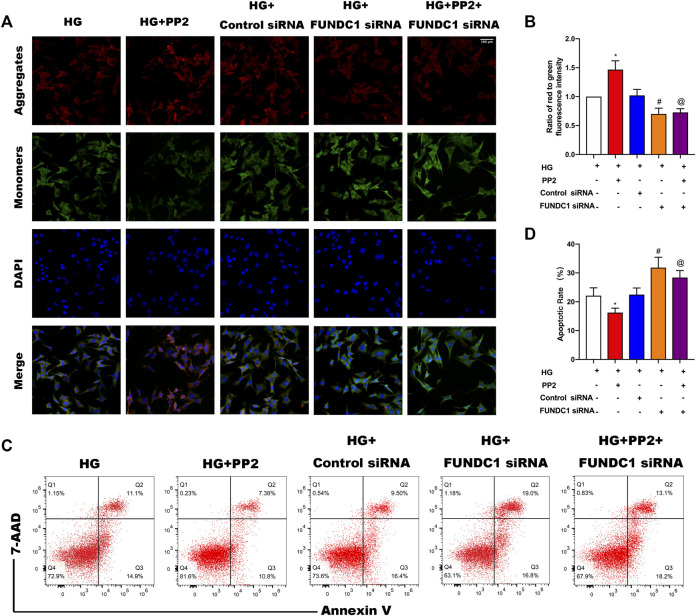
Silencing of FUNDC1 eliminates the protective effects of PP2 on podocytes exposed to HG conditions. **(A)** JC-1 staining to detect MMP. (Magnification, 200 ×). **(B)** MMP measured as the ratio of red to green fluorescence intensity from **(A)**. **(C)** Flow cytometry analysis of apoptosis in podocytes after the indicated treatments. **(D)** Quantitation of podocyte apoptosis from **(C)**. Values are expressed as the mean ± SD, *p < 0.05 vs. HG group, ^#^ *p* < 0.05 vs. HG + Control siRNA group, ^@^ *p* < 0.05 vs. HG + PP2 group.

Stimulation of podocytes with HG resulted in the collapse of mitochondrial membrane potential (Figures 7A,B), induction of podocyte apoptosis (Figures 7C,D). As expected from prior experiments, PP2 treatment alleviated the HG-induced changes. Interestingly, PP2 was unable to protect podocytes after transfection with FUNDC1 siRNA. Furthermore, FUNDC1-depleted podocytes were more susceptible to HG-mediated mitochondrial damage, apoptosis induction than control siRNA transfected podocytes. These data establish that FUNDC1 plays a protective role in preventing podocyte injury under HG conditions.

## Discussion

Previous studies have shown that mitophagy plays an important role in maintaining the health of the mitochondrial network (Killackey et al., 2020). Although mitophagy is closely related to the occurrence and development of DN (Yang et al., 2020), the relevant mechanisms still need to be further explored. In this study, we addressed the notion that Src pathway activation was linked to the inhibition of FUNDC1-mediated mitophagy during DN. Mechanistically, we found that activation of Src, as measured by its phosphorylation, can promote the inactivation of FUNDC1-mediated mitophagy *via* phosphorylation of FUNDC1. Moreover, evidence for this mechanism was obtained in both animal and cell models of DN. To our best knowledge, this is the first report to divulge the essential role of Src in regulating FUNDC1-mediated mitophagy in DN.

Src is a member of the non-receptor tyrosine kinase family. Two principles tyrosine residues are involved in the regulation of Src, one as activator and the other as an inhibitor. In Src, phosphorylation of Tyr416 promotes kinase activity while phosphorylation at Tyr527 causes inactivation. Src activity has been closely associated with the occurrence and development of chronic kidney diseases, including glomerulonephritis (Mima et al., 2011), polycystic kidney disease (Elliott et al., 2011), HIV-associated nephropathy (He et al., 2004) and diabetic nephropathy (Taniguchi et al., 2013). Regarding the latter, a large number of studies have shown that Src promotes the pathogenesis of DN through a variety of different mechanisms. These include effects on the proliferation of mesangial cells, apoptosis of renal tubular cells, and the activation of other pro-fibrotic signalling pathways including EGFR (Taniguchi et al., 2013), caveolin-1/RhoA (Lu et al., 2016), MAPK (Wu et al., 2015) and AKT (Choudhury et al., 2006). Although ample evidence has confirmed the negative effects of Src activation on DN, its detailed molecular machinery associated with mitochondrial physiology remains largely uncovered. Consistent with previous research (Taniguchi et al., 2013; Wu et al., 2015), we found that high glucose can increase Src Tyr416 phosphorylation levels in podocytes in a mouse model of type 2 diabetes. We further observed that Src inhibitor treatment contributed to lower blood glucose and creatinine levels and reduced the incidence of podocyte apoptosis. This result further establishes the principle that restraining Src activation may be a beneficial strategy for diabetes treatment through protecting podocytes from damage.

Podocytes are high energy-consuming cells, and their function strongly relies on mitochondria to provide energy (Denhez et al., 2019). Ample evidence demonstrates that mitochondrial dysfunction is one of the causes of diabetic nephropathy (Qi et al., 2017; Sorrentino et al., 2018) and consequently, the protective role of mitophagy to remove damaged mitochondria is highly significant in this context. Consistent with our findings, there is also increasing evidence confirming links between decreased mitophagy in both type 1 diabetic nephropathy and type 2 diabetic nephropathy (Li et al., 2017; Xiao et al., 2017; Zhou et al., 2019). The mechanisms involved have not been generally well studied except for PINK1-mediated mitophagy which plays a protective role in diabetic kidney disease (Wang et al., 2021). Moreover, activating the PINK1-Parkin pathway has been suggested as a beneficial approach to restore mitophagy (Huang et al., 2021). Our description of the regulatory mechanism involving FUNDC1-mediated mitophagy in models of type 2 diabetes offers new perspectives on the pathogenesis of DN.

FUNDC1-mediated mitophagy is a form of receptor-mediated mitophagy, which is regulated by reversible protein phosphorylation (Liu et al., 2014). Phosphorylated FUNDC1 creates steric hindrance for the binding of LC3II, thereby effectively inhibiting mitophagy (Zhang et al., 2016). Our results show that activated Src induced FUNDC1 Tyr18 phosphorylation which inhibited mitophagy in the context of DN, resulting in the inability of podocytes to clear damaged mitochondria. However, our results clearly establish that inhibiting Src activity can increase mitophagy in podocytes and reverse the deleterious effects of high glucose. Moreover, FUNDC1 knockdown experiments undertaken in cultured podocytes established that FUNDC1 mitophagy was, to a major extent, the downstream target of Src in DN. In addition to animals and cells, it will be also important to establish the relative importance of the Src-FUNDC1-mitophagy axis in clinical samples of DN and other degenerative kidney diseases.

**FIGURE 8:**
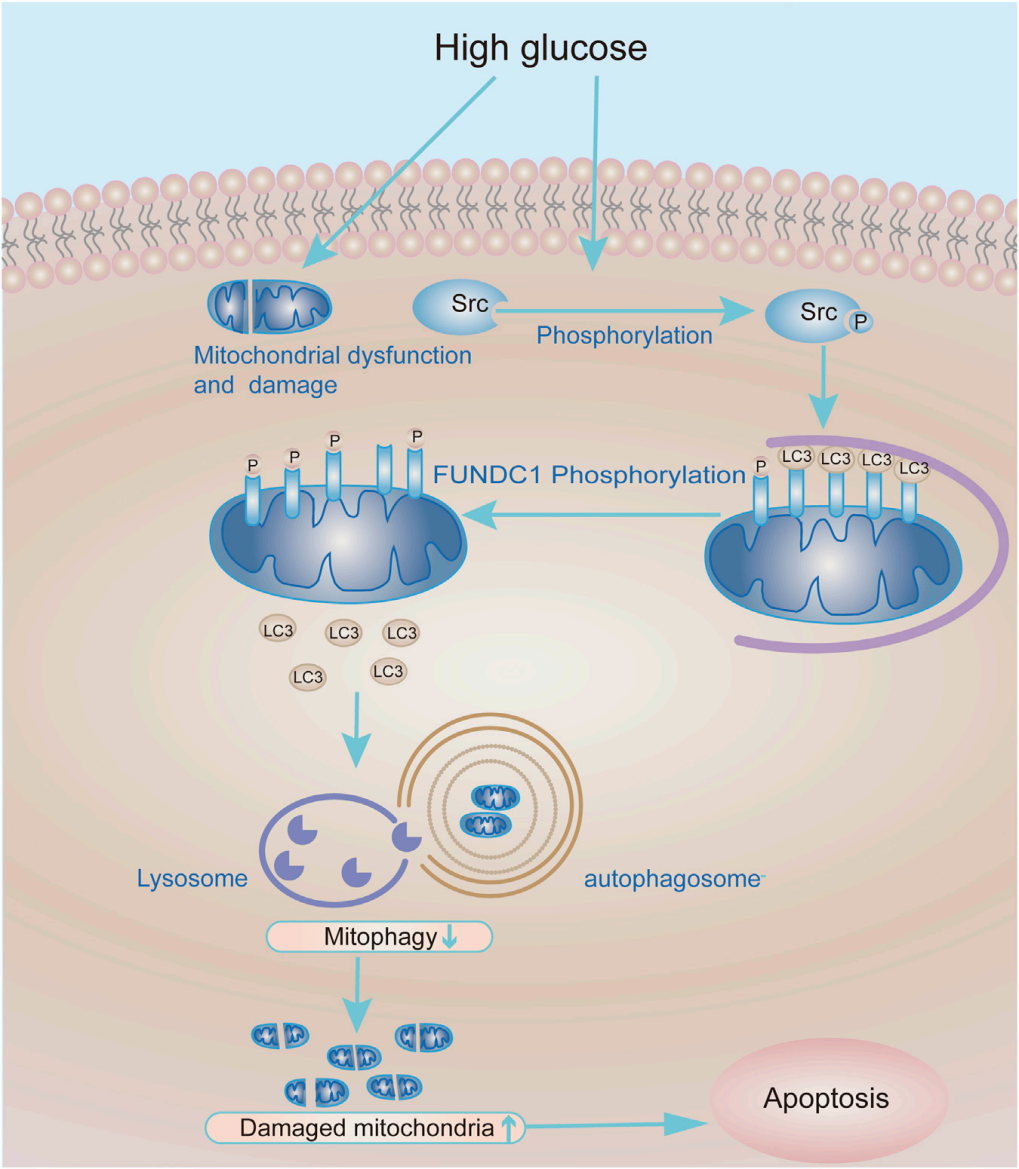
Working model of the molecular mechanism whereby Src inhibits mitophagy under HG conditions to cause podocyte injury. Under HG conditions, the expression and activity of Src are up-regulated in podocytes, leading to increased phosphorylation and inactivation of FUNDC1. In turn, this reduces the recruitment of LC3II to mitochondria and decreases mitophagy. Accumulation of damaged mitochondria in podocytes compromises their function and leads to podocyte apoptosis.

In summary, our research shows that inhibition of Src reactivates FUNDC1-mediated mitophagy in cell and animal models of DN. In consequence, mitophagy promotes podocyte survival and protects kidneys from the hyperglycaemic injury. Therefore, the Src-FUNDC1-mitophagy axis represents a potential therapeutic target in DN (Figure 8).

## Data Availability

The original contributions presented in the study are included in the article/Supplementary Material, further inquiries can be directed to the corresponding author.
