# Supplementary material for: Src Activation Aggravates Podocyte Injury in Diabetic Nephropathy via Suppression of FUNDC1-Mediated Mitophagy
Source: Front Pharmacol. 2022 May 9;13:897046. doi: 10.3389/fphar.2022.897046 (PMC9124787; doi:10.3389/fphar.2022.897046)

**Supplemental Material**

**Src activation aggravates podocyte injury in diabetic nephropathy via suppression of FUNDC1-mediated mitophagy**

Ting Zheng^1^, Han-yu Wang^1^, Yang Chen ^1^, Xiao Chen^1^, Zi-ling Wu^1^, Qin-yu Hu^1^, Hui Sun^1, *^

^1^ Department of Endocrinology, Union Hospital, Tongji Medical College, Huazhong University of Science and Technology, Wuhan, China

*** Correspondence:**Hui Sun
sunny68@hust.edu.cn

**Supplementary Table 1. Antibodies used in this study.**

| Primary antibodies | Host | Dilution and supplier | Application |
| --- | --- | --- | --- |
| Src | Rabbit | 1:1000; Cell Signaling Technology | WB |
| p-Src(Tyr416) | Rabbit | 1:1000; Cell Signaling Technology | WB |
| Nephrin | Guinea pig | 1:100; Progen, Germany | IF |
| FUNDC1 | Rabbit | 1:500; abcepta, SuZhou, China | WB |
| p-FUNDC1 | Rabbit | 1:500; abcepta, SuZhou, China | WB |
| LC3 | Rabbit | 1:1000; Proteintech, China | WB |
| P62 | Rabbit | 1:1000; Cell Signaling Technology | WB |
| LC3 | Rabbit | 1:200; Abcam | IF |
| TOMM20 | Mouse | 1:100; Santa Cruz Biotechnology | IF |
| GAPDH | Mouse | 1:3000; Antgene, China | WB |

**Supplementary Figure1**


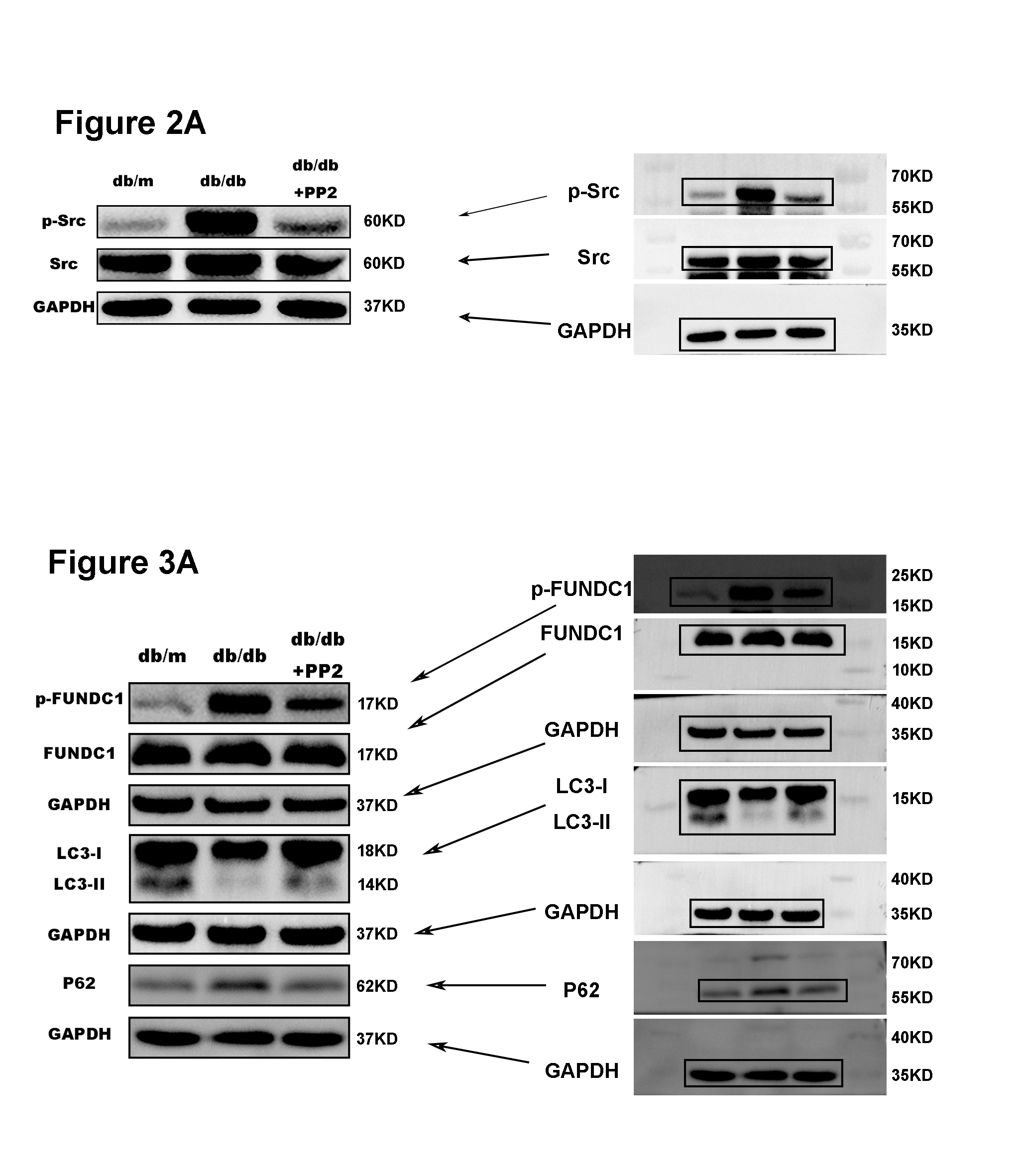

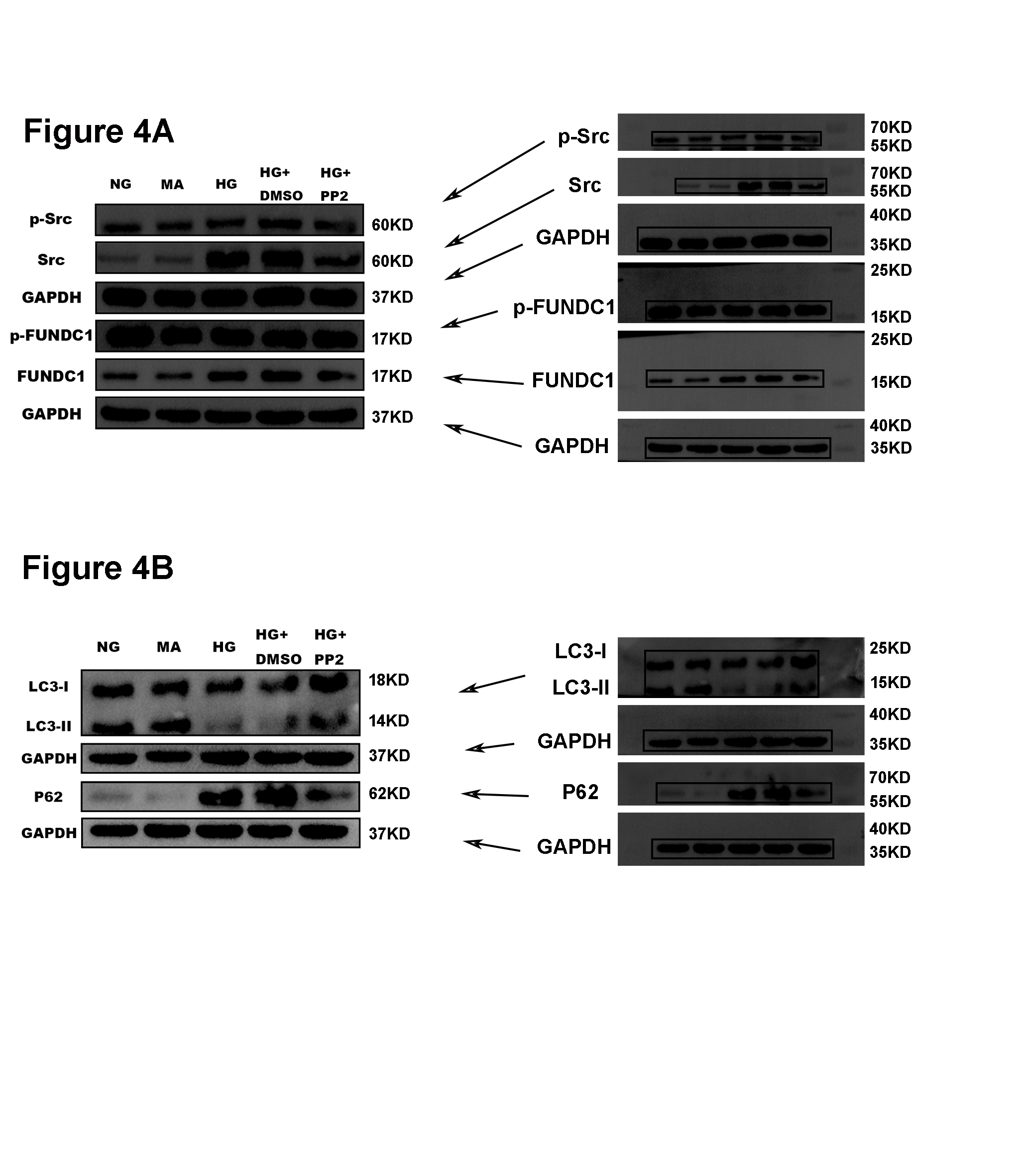


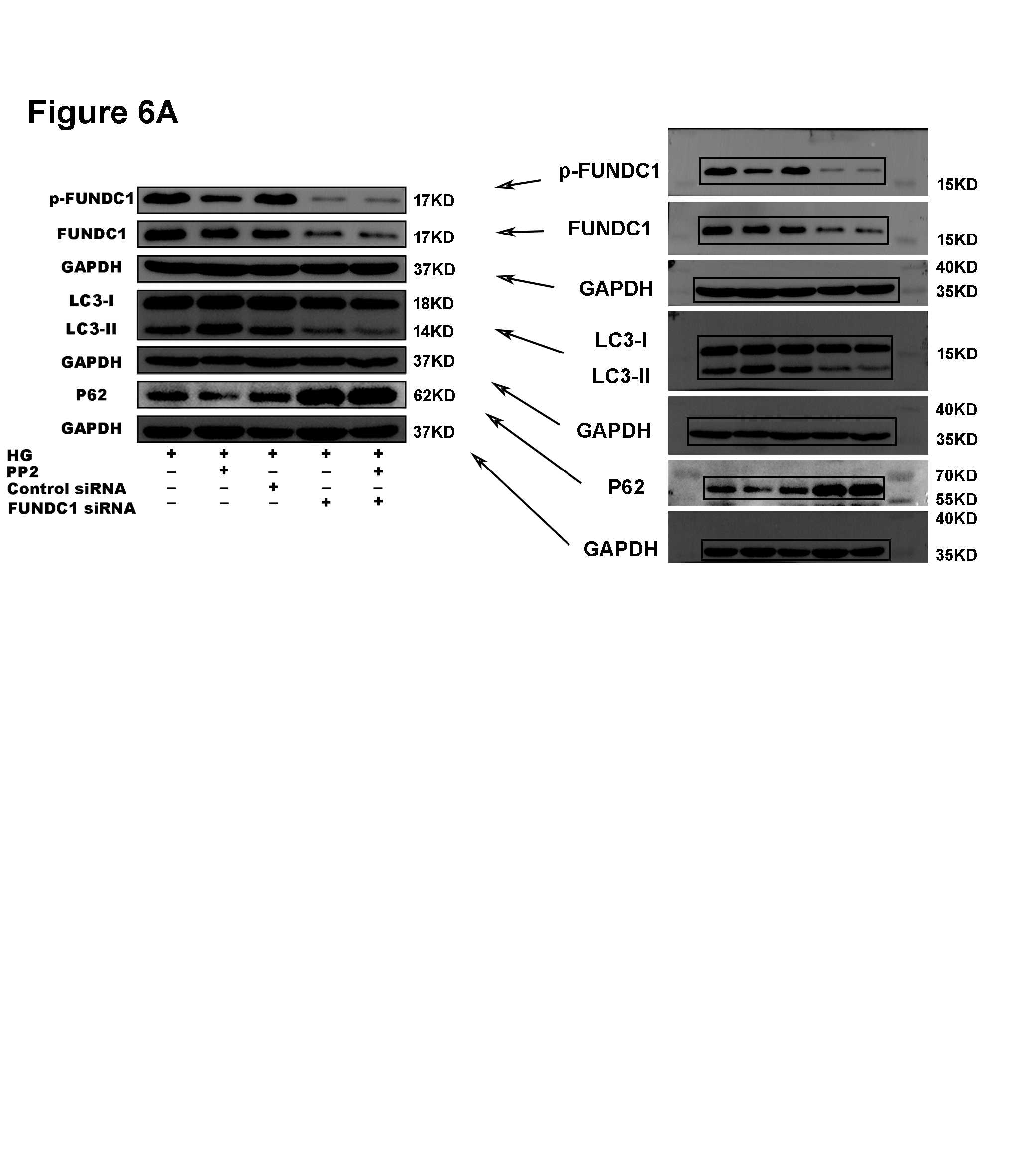

Supplement: Supplementary file 1 [file DataSheet1.DOCX]
